# Increased risk of low birth weight in women with placental malaria associated with *P. falciparum* VAR2CSA clade

**DOI:** 10.1038/s41598-017-04737-y

**Published:** 2017-08-11

**Authors:** Jaymin C. Patel, Nicholas J. Hathaway, Christian M. Parobek, Kyaw L. Thwai, Mwayiwawo Madanitsa, Carole Khairallah, Linda Kalilani-Phiri, Victor Mwapasa, Achille Massougbodji, Nadine Fievet, Jeffery A. Bailey, Feiko O. ter Kuile, Philippe Deloron, Stephanie M. Engel, Steve M. Taylor, Jonathan J. Juliano, Nicaise Tuikue Ndam, Steven R. Meshnick

**Affiliations:** 1 Department of Epidemiology, Gillings School of Global Public Health, University of North Carolina, Chapel Hill, USA; 2 Program in Bioinformatics and Integrative Biology, University of Massachusetts, Worcester, MA USA; 3 Curriculum in Genetics and Molecular Biology, University of North Carolina, Chapel Hill, USA; 4 College of Medicine, University of Malawi, Blantyre, Malawi; 5 Department of Clinical Sciences, Liverpool School of Tropical Medicine, Liverpool, United Kingdom; 6 Centre d’Etude et de Recherche sur le paludisme associé à la Grossesse et à l’Enfance, Université d’Abomey-Calavi, Cotonou, Benin; 7 COMUE Sorbonne Paris Cité, Université Paris Descartes, Paris, France; 8 UMR216 - MERIT, Institut de Recherche pour le Développement, Paris, France; 9 Division of Transfusion Medicine, Department of Medicine, University of Massachusetts, Worcester, MA USA; 10 Division of Infectious Diseases, Duke University Medical Center and Duke Global Health Institute, Durham, NC USA; 11 Division of Infectious Diseases, University of North Carolina School of Medicine, Chapel Hill, NC USA

## Abstract

Pregnancy associated malaria (PAM) causes adverse pregnancy and birth outcomes owing to *Plasmodium falciparum* accumulation in the placenta. Placental accumulation is mediated by *P. falciparum* protein VAR2CSA, a leading PAM-specific vaccine target. The extent of its antigen diversity and impact on clinical outcomes remain poorly understood. Through amplicon deep-sequencing placental malaria samples from women in Malawi and Benin, we assessed sequence diversity of VAR2CSA’s ID1-DBL2x region, containing putative vaccine targets and estimated associations of specific clades with adverse birth outcomes. Overall, *var2csa* diversity was high and haplotypes subdivided into five clades, the largest two defined by homology to parasites strains, 3D7 or FCR3. Across both cohorts, compared to women infected with only FCR3-like variants, women infected with only 3D7-like variants delivered infants with lower birthweight (difference: −267.99 g; 95% Confidence Interval [CI]: −466.43 g,−69.55 g) and higher odds of low birthweight (<2500 g) (Odds Ratio [OR] 5.41; 95% CI:0.99,29.52) and small-for-gestational-age (OR: 3.65; 95% CI: 1.01,13.38). In two distinct malaria-endemic African settings, parasites harboring 3D7-like variants of VAR2CSA were associated with worse birth outcomes, supporting differential effects of infection with specific parasite strains. The immense diversity coupled with differential clinical effects of this diversity suggest that an effective VAR2CSA-based vaccine may require multivalent activity.

## Introduction

Pregnant women are at increased risk of *Plasmodium falciparum* malaria infection and its adverse outcomes. Globally, an estimated 125 million pregnant women are at risk of malaria with 56 million of them living in regions with stable malaria transmission^1–3^. In these areas, pregnancy associated malaria (PAM) poses a significant risk to not only the mother but also to the newborn and is a common cause of adverse pregnancy and birth outcomes, including maternal anemia, stillbirth, preterm birth, and low birth weight (LBW). Additionally, PAM is estimated to result in 75,000–200,000 infant deaths annually^2–9^.

The adverse effects of malaria during pregnancy are in part the result of the ability of infected erythrocytes (IE) to accumulate in the placenta^10, 11^. This accumulation of IE in the placenta is facilitated by VAR2CSA, a specific variant of *Plasmodium falciparum* erythrocyte membrane protein 1 (PfEMP1). VAR2CSA is expressed on the surface of the IE and binds to the placental chondroitin sulfate A (CSA) on the syncytiotrophoblast^12, 13^. Immunity directed against VAR2CSA develops naturally with successive pregnancies and appears to protect against adverse birth outcomes^11^. Therefore, vaccination against VAR2CSA could be an effective way to reduce the effect of adverse outcomes from PAM^14–18^.

**Figure 1:**
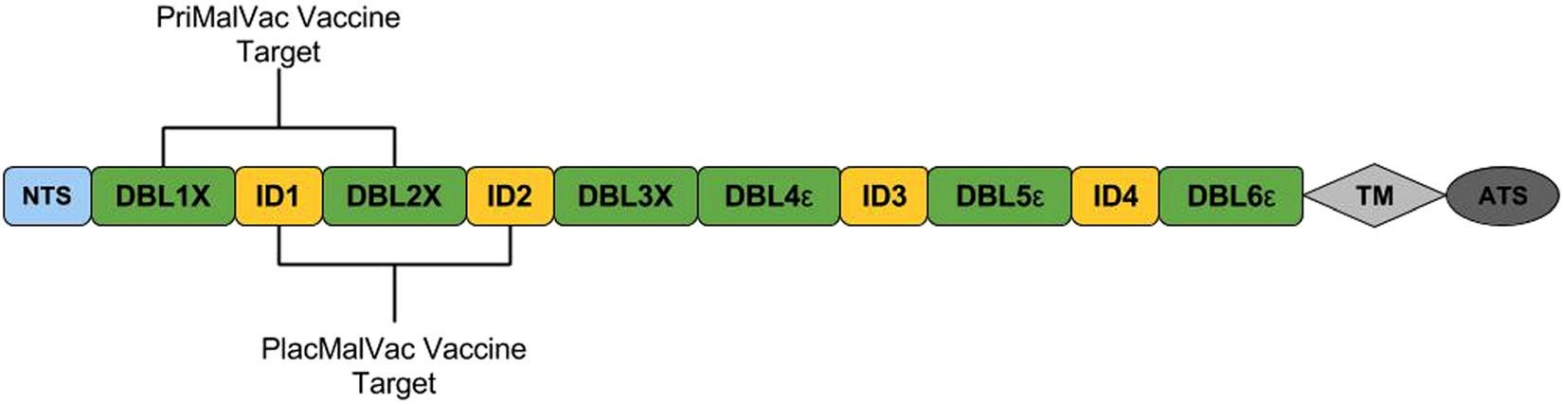
Schematic representation of the *var2csa* gene. VAR2CSA consists of an N-terminal segment (NTS), six Duffy Binding Like Domains (DBL), four cysteine-rich interdomain regions (ID), a transmembrane domain (TM), and an intra-cellular acidic terminal segment (ATS). Current vaccine development efforts (PlacMalVac and PriMalVac) are targeting the NTS-DBL2x region. The ID1-DBL2x region has recently been identified as the minimal CSA-binding region that contains major protective epitopes and elicits a strong host immune response^15, 21–29^.

VAR2CSA is a large 350 kDa transmembrane protein consisting of six Duffy binding like (DBL) domains and four interdomain (ID) regions (Fig. 1)^19, 20^. Several domains mediate the binding of IE to the CSA or elicit a host immune response^15, 17, 21–29^, and vaccine development efforts against PAM have been undermined in part because of the difficulty of producing a vaccine targeting such a large and diverse protein. However, the N-terminus region of VAR2CSA up to the DBL2x domain has been identified as critical to the binding process and able to induce antibody response with similar inhibitory capacity as that elicited against full-length VAR2CSA^30^. Recently, a small fragment of VAR2CSA within the N-terminus region, comprising the ID1-DBL2x region plus 93 amino acids of the ID2 interdomain region, was identified as the minimal CSA-binding region, and antibodies to this region abrogated the adhesion of IE to CSA *in vitro* ^31–34^. Therefore, the two leading PAM vaccine candidates, PlacMalVac and PriMalVac, each use a single recombinant protein that target overlapping constructs of this region (Fig. 1) but contain haplotypes from different parasite strains, either from strain FCR3 (PlacMalVac) or from strain 3D7 (PriMalVac)^35, 36^.

In the past, monovalent malaria vaccines have inadequately accounted for standing parasite antigen diversity, resulting in suboptimal efficacy and effectiveness, and a similar phenomenon has been observed *in vitro* with VAR2CSA-based candidate vaccines^37–40^. Therefore, a VAR2CSA-based vaccine against PAM may need to protect against many VAR2CSA strains to be effective. Currently, there is a paucity of data on the genetic diversity of ID1-DBL2x in field isolates; most prior genetic studies on VAR2CSA have either genotyped laboratory reference strains or only small gene fragments^21, 22, 31, 41–44^. A better understanding on the genetics and epidemiology of ID1-DBL2x antigenic diversity could inform vaccine development efforts.

**Figure 2:**
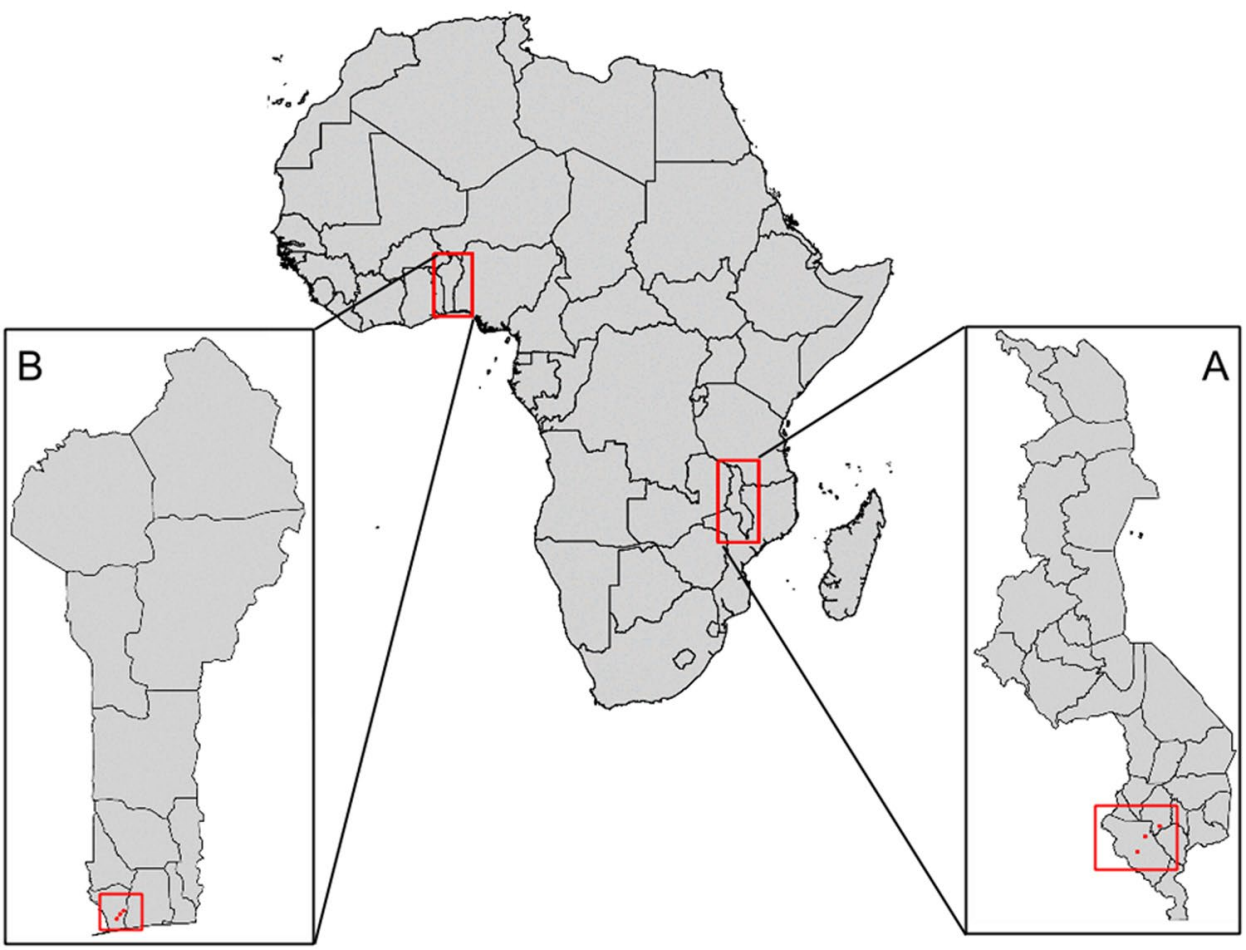
Study sites in (**A**) Malawi and (**B**) Benin. The map was created with RStudio, version 0.98.1083, https://www.rstudio.com/.

The aim of this study was two-fold: 1) to investigate the natural antigenic diversity of the ID1-DBL2x region of VAR2CSA and 2) to investigate the association between clades of the ID1-DBL2x region of VAR2CSA and adverse birth outcomes. Because the sequence and structural diversity of non-VAR2CSA *var* genes mediates severe malaria syndromes including cerebral malaria we hypothesized that VAR2CSA sequence diversity would mediate the severity of placental malaria^45^. Using a new long-read next-generation amplicon sequencing approach, we characterized a 1.6 kb region of ID1-DBL2x and assessed diversity among *P. falciparum* infected placental isolates from two previously completed studies in Malawi (n = 56) and Benin (n = 45) (Fig. 2). We then used phylogenetic methods to determine how different strains divided into clades. Lastly, we estimated the association of specific clades on birth weight, low birth weight (LBW), and small for gestational age (SGA), controlling for confounders using inverse probability weights. We found a high diversity of VAR2CSA within and between countries, but all parasites fell into one of five clades. Infection with parasites from different clades was associated with different risk of adverse infant outcomes.

## Results

### Participant Characteristics

We successfully amplified the 1.6 kb ID1-DBL2x region of *var2csa* in 101 of the available *P*. falciparum infected placental samples – 56/281 (19.9%) from Malawi and 45/126 (35.7%) from Benin, constituting the analytic population. Differences in amplification efficiency between study sites likely represent different sample types and sample storage. Among the available *P. falciparum* positive specimens, there were no significant differences in demographic characteristics between women whose samples amplified successfully and those whose samples did not amplify (Table S3). Demographic and clinical characteristics of the analytic population are shown in Table 1. Between countries, there were significant differences in maternal age (mean (standard deviation [SD]) 20.5 years [4.6] in Malawi versus 24 years [6.3] in Benin; p value = 0.013) and gestational age at delivery (mean [SD] 37.1 weeks [3.4] in Malawi versus 39.2 weeks [1.6] in Benin; p value < 0.001). There were also a significantly higher proportion of primiparous women in Malawi than Benin (51.8% versus 22.2%; p value = 0.004). The mean [SD] infant birthweight was similar in Malawi: (2677.7 g [539.6 g]) and Benin (2840 g [379.7 g]) (p value = 0.146). The prevalence of LBW was 19.6% (n = 11) in Malawi and 13.3% (n = 6) in Benin and prevalence of SGA was 16.1% (n = 9) in Malawi and 24.4% (n = 11) in Benin. There were no significant differences in the adverse birth outcomes, LBW or SGA by country (Table 1)

**Table 1:** Demographic and clinical characteristics of the analytic population.

	Malawi (n = 56)	Benin (n = 45)	p value
**Maternal Age (mean, (SD))**	20.5 (4.6)	24.0 (6.3)	0.013
**Gestational Age (mean, (SD))**	37.1 (3.4)	39.2 (1.6)	<0.001
**Parity (n, (%))**			
**Primiparous**	29 (51.8)	10 (22.2)	0.004
**Multiparous**	27 (48.2)	35 (77.8)	
**Birthweight (mean, (SD))**	2677.7 (539.6)	2840 (379.7)	0.146
**Low birthweight (LBW), n(%)**	11 (19.6)	6 (13.3)	0.459
**Small-for-Gestational-Age (SGA), n(%)**	9 (16.1)	11 (24.4)	0.535

### Validation of Amplification and Deep Sequencing

To ensure our PCR assay and clustering algorithm were valid, seven reference parasite lines mixed in five pools at varying frequencies were subjected to the amplification and analyses pipeline. In each of the five distinct multiclonal template mixtures, we accurately detected each of the seven parasite genomic lines (Figure S2). Additionally, we detected parasite haplotypes that were included in as little as 5% of the mixed template. Quantitatively, the observed frequencies of each parasite haplotype correlated well with the expected frequencies (R^2^ = 0.82) (Figure S3). The clustering of variants using the k-mer algorithm produced similar results as expected based on mixtures and highly similar to the reference-guided clustering. Some of the variation from the expected is likely due to inaccuracies of DNA quantification in the preparation of the mixing based on variation in DNA quantification methods (See Supplementary Information).

### ID1-DBL2x deep-sequencing among clinical isolates

The 101 clinical *P. falciparum* placental parasitemias were sequenced on 14 PacBio SMRT cells and collectively yielded a total 523,482 CCS reads. After extracting reads by barcoded primer sequences and filtering based on size and quality requirements, 256,377 (48.98%) reads were input into k-mer clustering to identify unique variants in our study population (Figure S4). The median sequence length from the extracted reads was 1594 bp (range: 1400–1700bp). The median coverage from CCS was 1795 reads per sample (range: 16–9241). Overall, k-mer clustering identified 152 unique ID1-DBL2x variants: 95 in Malawi and 57 in Benin. Of these 152 variants, 17 were observed in more than one woman, and only one variant was shared between Malawi and Benin. The mean number of ID1-DBL2x variants per women was 1.88 (range: 1–7) in Malawi and 1.56 (range: 1–4) in Benin. Overall the ID1-DBL2x fragment showed significant genetic variation, with ~30% of the nucleotide positions being polymorphic (Table S4).

**Figure 3:**
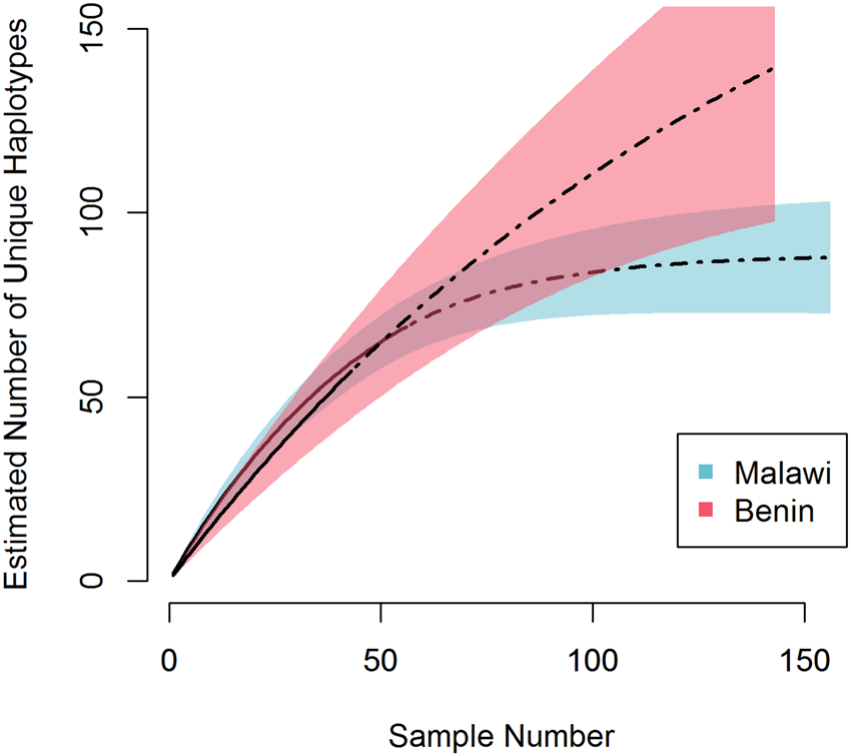
Rarefaction curves of ID1-DBL2x variants in Malawi (blue) and Benin (red). Calculated rarefaction curves are represented by solid lines. Extrapolated curves are depicted by dotted lines. The rarefaction curves were bootstrapped 1000 times to generate the 95% confidence intervals (CIs), which are represented by lightly shaded red and blue.

The within-population diversity of ID1-DBL2x at the two study sites was assessed by rarefaction, which compensates for the difference in depth of sampling (Fig. 3). The two curves overlap for most of the sampled region but only the extrapolated curve for Malawi approaches its asymptote, suggesting that ID1-DBL2x is more diverse in Benin as compared to Malawi. This finding is supported by other diversity ecologic metrics (Table S5) and by *H*_*e*_ at the amino acid level, where the ID1 region in Benin is more diverse than in Malawi (Figure S5). Between populations, Wright’s Fixation index (*F*_*ST*_) was estimated with a sliding window over the entire 1.6 kb region for pairwise comparison between countries, women with differing gravidities, and the two vaccine clades (3D7 and FCR3). Both the *F*_*ST*_ values comparing variants from women with differing gravidities and the values between countries approached zero (*F*_*ST*_ range: 0–0.11), indicating high degree of genetic similarity However, comparing *F*_*ST*_ values between 3D7 and FCR3 clade, overall the two clades look similar except for a ~100 bp region where the two populations differ significantly (*F*_*ST*_ = 0.26–1.0) (Figure S6). Assessment of Tajima’s D, a signature of balancing selection, shows similar evidence of immune selection across populations but with differences in the domains under selection (Table S4 and Figure S7).

### Phylogenetic clustering

**Figure 4:**
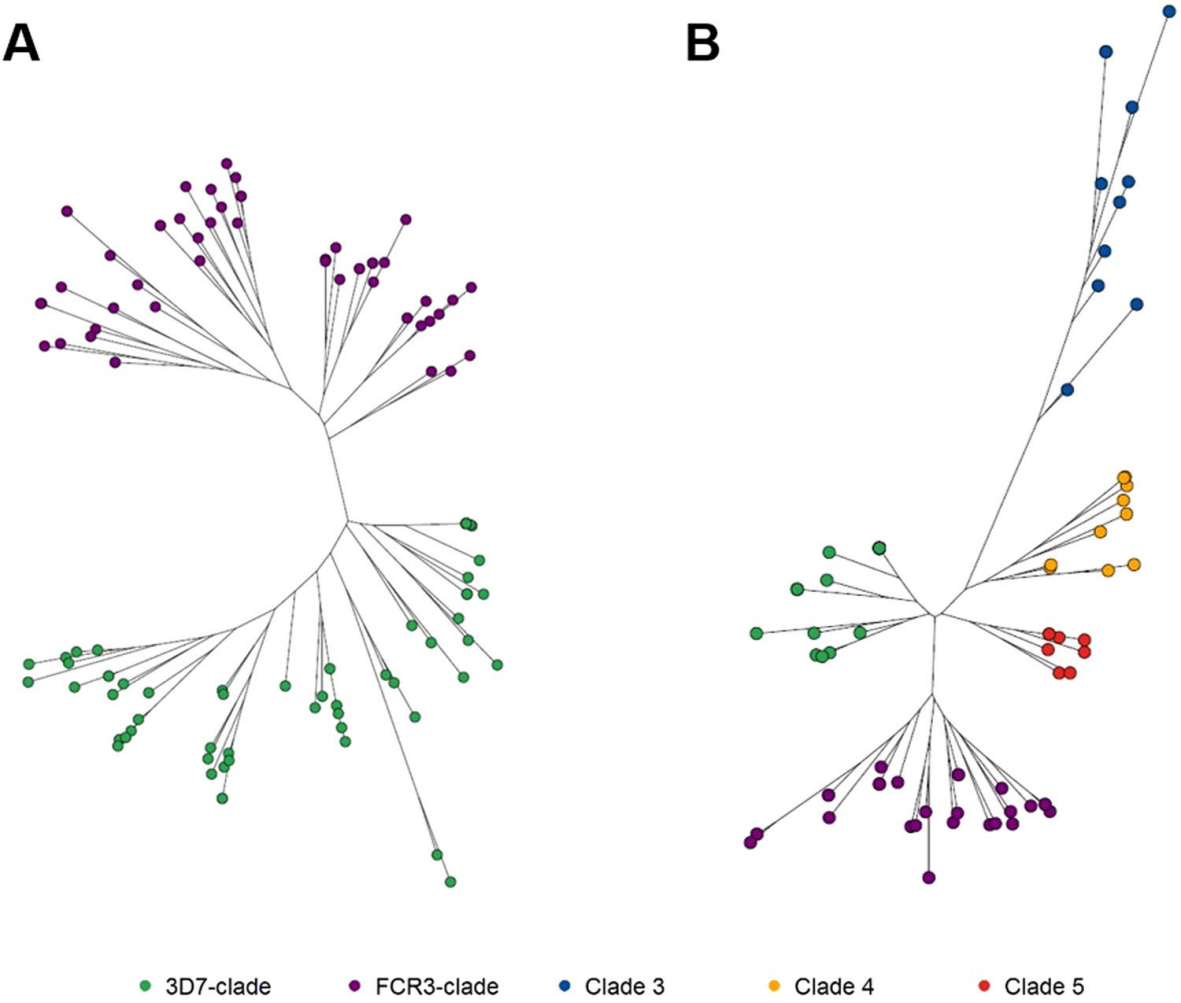
Maximum-likelihood phylogenetic trees of ID1-DBL2x variants in (**A**) Malawi and (**B**) Benin. All trees were bootstrapped 1000x and bootstrap values >80 were used as cutoff points for significant branch differentiation. In Malawi, variants clustered in two distinct clades with the two referent strains (3D7 and FCR3). In Benin, in addition to the 3D7 and FCR3 clades, three other clades were detected.

The maximum-likelihood phylogenetic trees clustered the observed variants from Malawi into two distinct clades, with the clustering supported by branch-point bootstrap values > 80%. The two clades co-clustered with the two referent strains which were used to design the current vaccines (3D7 or FCR3) (Fig. 4A). In Malawi, 21 (37.5%) and 22 (39.3%) women were infected with only 3D7-like and FCR3-like variants, respectively (Table 2). Variants from both clades were detected in 13 (23.2%) women. In contrast, the phylogenetic trees in Benin clustered the variants into five significantly distinct clades (bootstrap values > 80%). In addition to the 3D7 and FCR3 clades identified in Malawi, three clades unique to Benin were detected (Fig. 4B). While 3D7-like variants only infected 4 women (8.9%) and FCR3-like variants infected 13 (28.9%), 17 women (37.8%) were infected with a variant from one of the three clades unique to Benin. We also observed 6 (13.3%) women infected with both 3D7-like and FCR3-like variants, 4 (8.9%) women were infected with FCR3-like variants and variants from the three Benin unique clades, and one (2.2%) woman who was infected with variants from the two vaccine clades as well as the other clades (Table 2). The PCoA analyses showed that out of the non-vaccine clades, clades 4 and 5 appear to be closely related to the 3D7-like clade. Interestingly, variants in clade 3 displayed a genetic background resembling both 3D7-like and FCR3-like clades, indicating a possible recombination event (Figure S8).

**Table 2:** Distribution of infant birth weight and adverse birth outcomes by ID1-DBL2x clades in Malawi and Benin.

Clades	Malawi				Benin			
	N	SGA (%)	LBW (%)	Birthweight (median (IQR))	N	SGA (%)	LBW (%)	Birthweight (median (IQR))
**3D7**	21	6 (28.1)	10 (47.6)	2450 (2150–2850)	4	2 (50.0)	1 (25.0)	2710 (2490–2825)
**FCR3**	22	2 (9.1)	1 (4.6)	2800 (2600–2960)	13	3 (23.1)	2 (15.4)	2720 (2580–3250)
**3D7** + **FCR3**	13	1 (7.7)		2850 (2700–3000)	6	2 (33.3)	1 (16.7)	2900 (2550–3200)
**Other**					17	3 (17.6)	1 (5.9)	3100 (2790–3160)
**FCR3** + **Other**					4		1 (25.0)	2550 (2365–2610)
**3D7** + **FCR3** + **Other**					1	1 (100)		2700

### Associations with birthweight

The median (IQR) infant birthweight among mothers in Malawi infected with only 3D7-like variants was 2450 g (2150 g–2850 g) whereas mothers infected with only FCR3-like variants had a median birthweight of 2800 g (2600 g–2960 g). In Benin, the median (IQR) infant birthweight of mothers infected with only 3D7-like variants was 2710 g (2490 g–2825 g) and 2720 g (2580 g–3250 g) for those women infected with only FCR3-like variants **(**Table 2).

In both crude and adjusted regression models, compared to mothers infected with variants only from the FCR3-like clade, mothers infected with variants from only 3D7-like clade delivered babies with lower infant birthweight (adjusted difference: −267.99 g; 95% CI: −466.43 g, −69.55 g) (Table 3). This reduction in infant birthweight was present when each country was analyzed alone: In Malawi, placental infection with 3D7-like variants was associated with a reduction in infant birthweight of 330.97 g (95% CI: −628.44 g, −33.49) and in Benin by 147.88 g (95% CI: −530.06, 234 g) after controlling for parity (Table 3), but the latter confidence intervals included 0.

**Table 3:** Associations between ID1-DBL2x clades and infant birthweight (grams).

Birthweight	Crude			Adjusted^*^		
	Effect^**^	95% CI		Effect^**^	95% CI	
**Pooled**						
**FCR3**	REF	—	—	REF	—	—
**3D7**	−251.49	−474.28	−28.69	−267.99	−466.43	−69.55
**3D7** + **FCR3**	99.55	−151.45	350.56	22.36	−199.23	243.94
**Malawi**						
**FCR3**	REF	—	—	REF	—	—
**3D7**	−262.24	−557.46	33.00	−330.97	−628.44	−33.49
**3D7** + **FCR3**	151.76	−204.90	508.43	100.75	−260.62	462.12
**Benin**						
**FCR3**	REF	—	—	REF	—	—
**3D7**	−153.41	−548.08	241.26	−147.66	−530.06	234.75
**3D7** + **FCR3**	14.10	−328.97	357.15	21.99	−310.39	354.39
**Other** ^†^	187.33	−74.23	448.89	185.63	−67.80	439.06
**FCR3** + **other** ^†^	−323.41	−718.08	71.26	−280.21	−662.62	102.19
**3D7** + **FCR3** + **other** ^†^	−110.91	−816.91	595.09	−133.24	−906.49	640.02

^*^Adjusted for country (pooled analysis only) and parity using inverse probability weights (IPW).

^**^Change in infant birth weight (grams).

^†^Other clade includes variants from clades identified only in Benin.

In Benin, women infected with variants from clades other than the vaccine clades delivered babies with increased infant birthweight (185.63 g; 95% CI: −67.80, 439.06 g) as compared to women infected with only FCR3-like variants. Similarly, women co-infected with both 3D7-like and FCR3-like variants delivered babies with increases in infant birthweight across all populations (Table 3). However, women infected with variants from both vaccine and non-vaccine clades reported significantly lower infant birthweight across both countries (range: −280g – −133.41 g) (Table 3).

### Association with small-for-gestational-age (SGA) and low birth weight (LBW)

**Figure 5:**
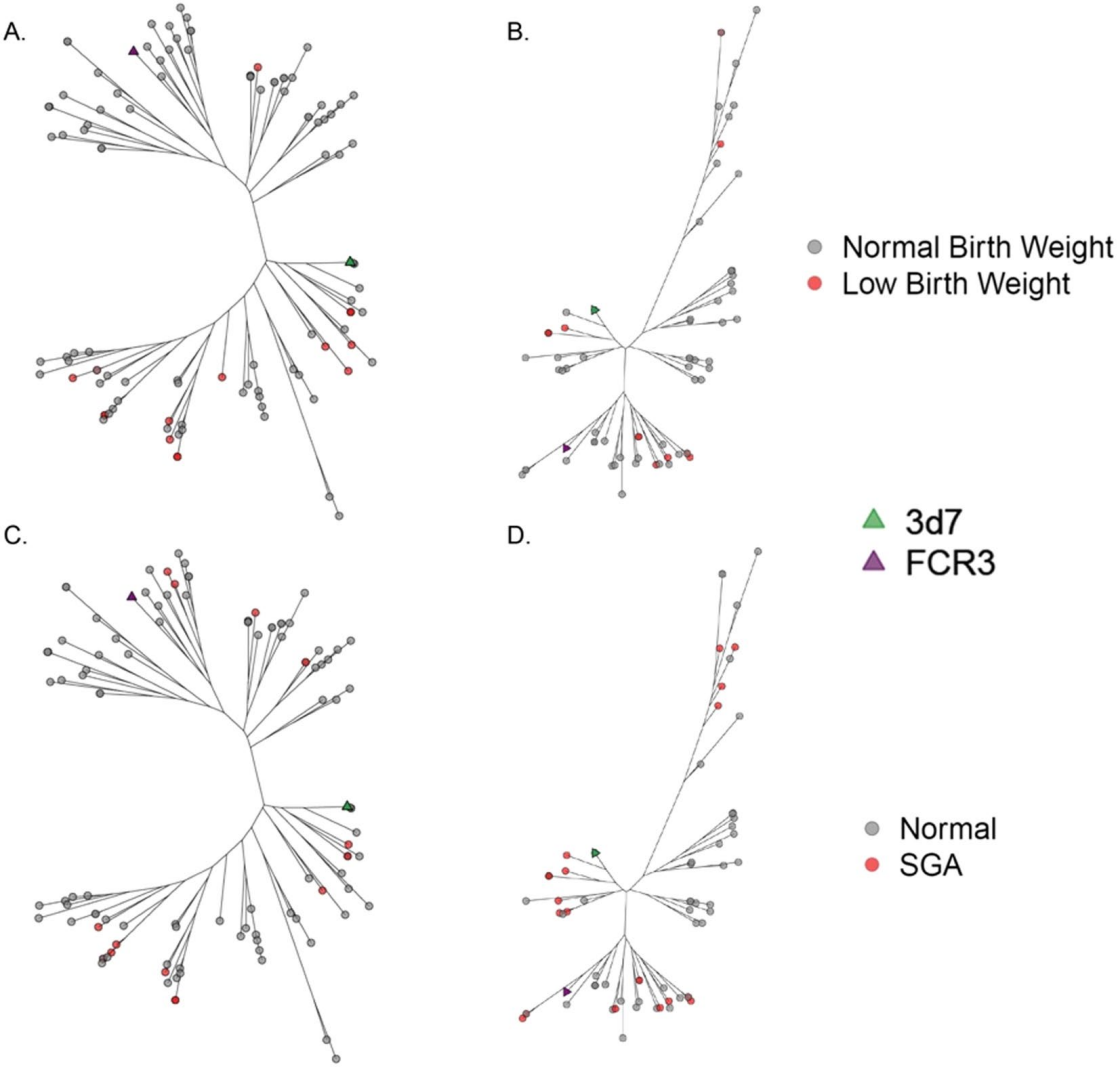
Maximum-likelihood phylogenetic trees of ID1-DBL2x variants by low birth weight (LBW) and small-for-gestational age (SGA). Panel A and C show data for Malawi for LBW and SGA, respectively. Panels B and D show data for Benin for LBW and SGA, respectively. All trees were bootstrapped 1000x and bootstrap values >80 were used as cutoff points for significant branch differentiation. LBW was defined according to World Health Organization’s criteria as infant with a birth weight of less than 2500 grams. 3D7 (green) and FCR3 (purple) were detected in both countries. Majority of the LBW variants (red) in Malawi were present in the 3D7-like clade (green) whereas in Benin, the LBW variants were more evenly distributed between 3D7-like (green) and FCR3-like (purple) clades. SGA was defined as infant with a birth weight below the 10^th^ percentile for babies of the same gestational age at delivery. 3D7 (green) and FCR3 (purple) were detected in both countries. SGA variants (red) clustered more readily in 3D7-like clade in Malawi whereas in Benin, the distribution was more even between 3D7-like and FCR3-like clades. We also identified four SGA variants clustered in clade 3.

Given the sparse distribution of categorical adverse birth outcomes SGA and LBW in our study population (Table 2), we modeled the effect of only the vaccine clades (3D7-like and FCR3-like) on SGA and LBW. Compared to women infected with only FCR3-like variants, the unadjusted prevalence of LBW was higher in women with only 3D7-like variants in Malawi (47.6% versus 4.6%) and in Benin (25% versus 15.4%) (Table 2). Phylogenetically, while there were a few LBW variants clustered in the FCR3-like clade, the majority of the LBW variants in Malawi were present in the 3D7-like clade (Fig. 5A). In Benin, distribution of LBW variants was more evenly distributed between 3D7-like and FCR3-like clade (Fig. 5B). In a pooled analysis adjusted for parity and country, compared with women infected only with FCR3-like variants, those infected with only 3D7-like variants had higher odds of LBW (OR: 5.41; 95% CI: 0.99, 29.52).

Similarly, the unadjusted prevalence of SGA was higher among women infected with only 3D7-like variants than women infected with only FCR3-like variants in Malawi (28.1% versus 9.1%) and in Benin (50% versus 23.1%) (Table 2). As observed with LBW, in pooled analysis, compared to women infected with only FCR3-like variants, women infected with only 3D7-like variants had higher odds of delivering a SGA infant after controlling for parity and country (OR 3.65; 95% CI 1.01–13.38) (Table S6). When stratifying by country, though not statistically significant, women infected with only 3D7-like variants had higher odds of delivering a SGA infant in Malawi (OR: 5.21; 95% CI: 0.77–35.41) and in Benin (OR: 2.98; 95% CI: 0.27–32.48) (Table S6).

## Discussion

A VAR2CSA-based vaccine against PAM will be the first syndrome-specific malaria vaccine and may substantially reduce one the major causes of adverse delivery outcomes in malaria-endemic sub-Saharan Africa. The current vaccine candidates targeting the N-terminus region of VAR2CSA are monovalent vaccines. Our study shows that the ID1-DBL2x epitope in the N-terminus region is highly diverse among *P*. falciparum-infected pregnant women in Malawi and Benin. There were 152 variants found among 101 women, and they clustered phylogenetically into five distinct clades. There was more genetic diversity in Benin than Malawi with sequence-based evidence of prior immune selection in both populations. Furthermore, variants from the 3D7-like clade were associated with adverse birth outcomes including newborns with lower birthweight. Taken together, the intrinsic diversity of this VAR2CSA region along with the association of a specific *P. falciparum* genotype with more severe clinical consequences suggest that an effective VAR2CSA-based vaccine aimed to prevent placental malaria and its sequelae will require multivalent activity.

The high degree of genetic diversity of *var2csa* observed in our study population is concordant with previously reported overall species richness of *var* genes^46^. In Malawi and Benin, among the 152 variants, only 17 were found in more than one woman and a single haplotype was shared between the two countries. Rarefaction curves show that the population in Benin, which contained all five clades, is more diverse than the population in Malawi, which only contained the 3D7-like and FCR3-like clades. Multiple ecological indices corroborate this difference in haplotype richness between sites. Several reasons may explain this higher diversity, including that transmission of malaria is higher in Benin. The high levels of diversity has important implications for future vaccine design. Taking the relevant genetic diversity into account when designing a vaccine will be critical in producing a candidate that has excellent cross-protective effect.

The primary driver for differentiation between clades was a 100 bp region that separated 3D7-like and FCR3-like parasites. This is seen by the high *F*_*ST*_ in the 100 bp region, and relatively low *F*_*ST*_ across the rest of the gene (Figure S6). Interestingly, all parasites, including those in the 3 clades unique to Benin, contained either a 3D7-like or FCR3-like domain. The branching off of these 3 additional clades were primary driven by differences in the ID1 region. This is consistent with our previous observation of an ID1 sequence of *var2csa* which appears to be unique to Benin^47^. Furthermore, some of the Beninese variants had large insertions in this domain.

Fortunately, our data also suggest that a ID1-DBL2x vaccine that is broadly effective in one country has a good chance to be successful in multiple countries. Between countries, there was almost no genetic differentiation detected by *F*_*ST*_ across the entire ID1-DBL2x region. Variants in the two countries were also more similar based on amino acid sequence than nucleotide sequence (Figure S5). This pattern of high overall nucleotide diversity, but a similar amino acid content has been described previously between Malawi and The Gambia for the antigen in the RTS,S vaccine, which showed similar efficacy in Malawi and West Africa^48^. Surprisingly, there was little genetic difference between primi-, secundi-, and multigravid women in terms of complexity of infection and the presence or absence of specific variants. We know that multigravidae develop immunity to VAR2CSA; these results suggest that the immunity may not be strain specific.

Importantly, we observed an association between specific *P. falciparum* clade and worsened birth outcomes. Overall, the 3D7-like clade was associated with adverse birth outcomes. Compared to women infected with only FCR3-like variants, women infected with variants only from 3D7-like clade delivered babies with reductions in birthweight between 147.7 g to 331.0 g in both Malawi and Benin, respectively (Table 3). This reduction in birth weight is clinically significant and comparable to the reduction in birth weight observed due to active maternal smoking – approximately a 200 g deficit^49^. Women infected with variants from only 3D7-like clade also had higher odds of delivering a LBW and SGA infant. Even though we used FCR3-like clade as a referent group to estimate relative pathogenicity, we observed that LBW and SGA variants indeed clustered in FCR3-like clade in both Malawi and Benin. The magnitude of the association between infection from 3D7-like variant and poor outcome was greater in Malawi than Benin. This could have been due to the fact that Benin experiences higher levels of malaria transmission than Malawi; pregnant women in Benin might have had greater pre-existing immunity than pregnant women in Malawi and this immunity could have mitigated strain-dependent pathogenicity.

Despite this association, adverse events were found within other clades. This suggests, that while a 3D7-like vaccine might be a preferable monovalent vaccine, a polyvalent or strain transcendent vaccine that protects against multiple clades would be more efficacious. While *in vivo* evidence of the strain specificity of immunity to this region of VAR2CSA is limited, there is some supportive evidence for its role. First, *in vitro* studies show a suboptimal cross-protective effect (between 3D7 and FCR3 clades) based on *in vitro* blocking assays^37, 41^. Second, we show that while overall the gene exhibits a strong genetic signature of immune selection based on Tajima’s D values of >1 (Table S3), the domains of elevated Tajima’s D vary across clade (Figure S7). This is similar to the genetic signal that has been seen at orthologous antigens between malaria species which are known to interact differently with host immunity^50^. Both of these lines of evidence suggest that the interaction with the host immune system is different between the clades and that immune selection is likely the primary driver for the diversity of the antigen.

Previous studies have relied on Sanger sequencing or cloning, both of which limit depth and sensitivity, or on breaking up the fragment into smaller fragments to deep-sequence and reassembling haplotypes *in silico*, a method which may complicate haplotype reconstruction^21, 22, 31, 41–44^. The PacBio CCS platform allowed us to sequence a long fragment of *var2csa* at great depth without requiring assembly of haplotypes. Additionally, barcoding individual samples allowed us to link variants detected through deep-sequencing to individual women and their clinical and demographic data. Due to the high rates of polymorphism, polyA repeats, and PacBio error rate, variant calling through conventional software was not adequate. As a result, we implemented a k-mer clustering algorithm which provided a more appropriate way to determine unique variants of *var2csa*.

Our study was limited by the number of *P*. falciparum infected placental samples we were able to include in our analyses. This was in part due to low yield in amplifying ID1-DBL2x, a long hypervariable region of *var2csa* from DNA extracted from dried blood spots in Malawi. The generalizability of our results is also limited as we had access to samples from only two countries in Africa. However, the results from our study agree with other studies that were able to deep-sequence smaller fragments of *var2csa* from multiple countries in Africa as well as from Asia and South America^31^. We also had sparse distribution of our categorical adverse birth outcomes that precluded us from detecting significant associations. However, including birth weight as a continuous outcome and controlling for confounders using inverse probability weights increased our ability estimate effect of vaccine clades on infant birth weight. Given that gestational age at delivery is a strong predictor of infant birth weight and is itself affected by malaria exposure during pregnancy, gestational age at delivery may be a mediator on the causal pathway of ID1-DBL2x clade and birth weight. We were therefore, limited in our ability to estimate the direct effect of ID1-DBL2x clade on birth weight. We were able to only estimate the total effect of ID1-DBL2x clade on birth weight, including the effect of gestational age on birth weight. This limited our ability to draw inferences on the effect of ID1-DBL2x clades on fetal growth specifically.

This is the first study investigating effects of specific genetic variants of *var2csa* on clinical outcomes. We employed an integrative approach, using molecular epidemiology, next-generation sequencing, and population genetics to examine the antigenic diversity of *var2csa*, identify immunologically relevant selection pressures, understand the genetic relatedness between populations, and identify ID1-DBL2x clades with differential pathogenicity. Previous attempts to develop a general vaccine against malaria have not taken parasite antigenic diversity into account; this is one possible explanation for suboptimal efficacy and effectiveness^38, 40^. The results from our study provide strong support for the development of polyvalent VAR2CSA-based vaccines against PAM. As clinical trials get underway for testing vaccines against PAM, characterizing genetic diversity and monitoring parasite populations will help explain changes that affect vaccine efficacy and inform future vaccine improvement and development efforts.

## Methods

### Study population and sample collection

Clinical samples from two previously completed studies in Malawi and Benin were used (Fig. 2). In Malawi, dried blood spot (DBS) samples of placental blood were obtained from a randomized controlled trial conducted between 2010 and 2013, which aimed to assess the efficacy of intermittent screening and treatment in pregnancy (ISTp) with Dihydroartemisinin-Piperaquine (DP)^51^. In Benin, packed red blood cell (pRBC) samples of placental blood were acquired from a prospective cohort study conducted between 2008 and 2010 to quantify the effects of PAM and investigate immunological responses to malaria infection during pregnancy^52^. All samples included in these analyses were placental blood specimens, collected at delivery from *P. falciparum*-infected pregnant women as determined by microscopy or RDTs and later tested by PCR targeting the *P. falciparum lactate dehydrogenase* (pfLDH) gene. At delivery, demographic and clinical data were collected on mothers and new-borns. Information regarding reproductive history, current pregnancy, and infant health was also collected. Additional details of the studies have been published elsewhere and is summarized in Supplementary Information.

Women in both studies were enrolled after obtaining signed informed consent. The current and parent studies were approved by institutional review boards at Institut de recherche pour le développement in France, Science and Health Faculty (University of Abomey Calavi) in Benin, Liverpool School of Tropical Medicine, Malawian National Health Science Research Committee, and University of North Carolina at Chapel Hill. All methods were carried out in accordance with the approved guidelines and regulations.

### Amplification of the ID1-DBL2x Domain

For Malawi specimens, genomic DNA (gDNA) was extracted from dried blood spots using 20% Chelex-100 (Bio-Rad, Richmond, CA) as previously described^53^. For Benin specimens, gDNA was extracted from RBC pellets using Thermo Scientific GeneJET DNA extraction kit (Thermo Fisher, Waltham, MA) as recommended by the manufacturer. From all gDNA, we then amplified the target region in technical duplicates using a hemi-nested PCR amplification strategy that used barcoded forward and reverse primers specific to each patient and technical replicate (Table S2). To inform primer design we used all publically available *var2csa* reference sequences to guide primer placement. Details about primer design and PCR conditions are provided in the Supplementary Information.

### Amplicon deep sequencing

Concentrations of successful PCR amplifications were quantified using the Genomic DNA ScreenTape assay on the Agilent 2200 TapeStation system. PCR products were then pooled together based on barcode compatibility. Each pool was gel extracted and purified using Qiagen gel extraction kit (Qiagen, Carlsbad, CA) per manufacturer’s protocol, quantified by Tapestation, and submitted for deep-sequencing on the PacBio circular consensus sequencing (CCS) platform using the P5-C4 chemistry at the UNC Highthrouput Sequencing Facility.

### Generation of ID1-DBL2x Haplotypes

Raw sequences were cleaned and clustered using a novel clustering algorithm based on k-mer distances^54^. The end result of k-mer clustering yielded one or more phased consensus sequences for each woman present in the study population. Haplotypes from technical PCR duplicates from each patient sample were only accepted when they appeared in both replicates. The haplotype frequencies were calculated as the average of the frequencies in the two replicates. All variants were initially aligned in MEGA6 using MUSCLE alignment^55^. Due to a large number of insertions and deletions and polyA repeats, we manually curated the alignment to improve the alignment output from MEGA6. Details of the clustering method are provided in the Supplementary Information and Supplementary Figure S1. All sequences have been submitted to GenBank (GenBank accession numbers: MF061812-MF061963).

### Validation of Haplotype Determination

We conducted a validation study using pools of reference genomic parasite lines to ensure that our assay and clustering algorithm were appropriate and accurate. We mixed seven reference parasite lines (3D7, FCR3, 7G8, DD2, K1, RO33, and V1/S) from BEI resources (formerly known as MR4, Manassas, VA) in varying frequencies (1–50%) in five pools and subjected them to the above amplification and analyses pipeline. Details of mixture generation are in the Supplementary Information. We estimated the component parasite lines for each pool and their relative frequency. We then compared the “expected” results to *de novo* clustering and reference-guided clustering (determined by aligning individual sequencing reads to the known reference sequence for each strain ascertained by primer walking and Sanger sequencing done at Eton Biosciences, RTP, NC) to determine “observed” haplotype frequencies.

### Within population diversity, between population diversity and phylogenetics of ID1-DBL2x

To better characterize the natural diversity of ID1-DBL2x, we assessed population diversity and the phylogenetics in order to assess the extent of antigen diversity, for sharing of haplotypes between sites, and for clustering of haplotypes into clades. A brief summary of approaches is provided here with additional details in the Supplementary Information. Measurements of within population (alpha) diversity were calculated in EstimateS^56^ and R using the adegenet and pegas packages^57, 58^. We determined bootstrapped rarefaction curves for each population, as well as heterozygosity (*H*_*e*_), Shannon index (H’), abundance coverage estimator (ACE), incidence coverage estimator (ICE), and Chao richness estimators^59–63^. We then compared these measures between the two countries and between women of different gravidity. Statistically significant differences in alpha diversity metrics between different groups were tested using Kruskal–Wallis test. An α of 0.05 was determined *a priori* to test for significant differences.

To characterize between population (beta) diversity, we assessed Wright’s fixation index (*F*_*ST*_) and performed a principal coordinate analysis (PCoA) to assess the genetic relatedness^64^. We calculated nucleotide diversity (π) and Tajima’s D test^65^ for the entire ID1-DBL2x region as well as with a sliding window approach to assess localized selection pressures acting upon vaccine target^66^. Tajima’s D was calculated on ID1-DBL2x variant populations by country and major clades. Differences were assessed between Malawi and Benin as well as between the different clades identified by phylogenetic analysis. All measures of genetic relatedness and selection (*F*_*ST*_, PCoA, π, Tajima’s D) were calculated in R using the adegenet^57^ and PopGenome^67^ packages.

Phylogenetic trees were generated in MEGA6 using the maximum composite likelihood method where genetic distances were calculated through the Tamura-Nei model^68^. The phylogenetic trees were bootstrapped 1000 times to compute branch support and provide precision in the clustering detected. Bootstrap values of greater than 80% were used as cutoff point to identify significant distinct clades. All phylogenetic trees were visualized using the APE package for R^69^.

### Associations of VAR2CSA clade with delivery outcomes

We examined associations between clades and birth outcomes to identify pathogenic clades. Outcomes of interest were infant birth weight, LBW, and SGA. At delivery, infant birth weight was recorded to the nearest gram within 24 hours using a calibrated digital scale. LBW was defined as a birth weight of less than 2500 grams^70^. SGA was defined as an infant with a birth weight below the 10^th^ percentile for babies of the same gestational age at delivery^71^.

Associations were determined by first examining if there was any clustering of LBW and SGA by clades in the MLE phylogenetic trees. Pregnant women were categorized based on the respective clade in which the variants detected from their samples clustered. We then used linear and logistic regression to estimate the effect of clades on birth weight, LBW, and SGA, respectively. We controlled for potential confounders and standardized the groups defined by clades to the marginal distribution of potential confounders in the study population using inverse probability weights. The denominator of the weights for the different clades of ID1-DBL2x was estimated using polytomous logistic regression, conditional on parity. For the pooled analyses, we also included country in estimating the denominator of the weights. Weights were stabilized using the marginal distribution of the ID1-DBL2x clades in our study population. We estimated differences in birth weight and relative odds of LBW and SGA associated with infection with each clade, using FCR3 as the referent category. All analyses were conducted using SAS version 9.3 (SAS Institute, Cary, North Carolina).

## Electronic supplementary material

Supplementary Information
