# Supplementary material for: Increased risk of low birth weight in women with placental malaria associated with P. falciparum VAR2CSA clade
Source: Sci Rep. 2017 Aug 11;7:7768. doi: 10.1038/s41598-017-04737-y (PMC5554196; doi:10.1038/s41598-017-04737-y)

**SUPPLEMENTARY INFORMATION (SI)**

# *Increased risk of low birth weight in women with placental malaria associated with P. falciparum VAR2CSA clade*

Jaymin C. Patel[1*]
Nicholas J. Hathaway[2]
Christian M. Parobek[3]
Kyaw L Thwai[1]
Mwayiwawo Madanitsa [4,5]
Carole Khairallah[5]
Linda Kalilani-Phiri [4]
Victor Mwapasa[4]
Achille Massougbodji[6]
Nadine Fievet[7,8]
Jeffery A. Bailey[9]
Feiko O ter Kuile[5]
Philippe Deloron[7,8]
Stephanie M. Engel[1]
Steve M. Taylor[1,10]
Jonathan J. Juliano[3,11]
Nicaise Tuikue Ndam[7,8]
Steven R. Meshnick[1]

[1] Department of Epidemiology, Gillings School of Global Public Health, University of North Carolina, Chapel Hill, USA
[2] Program in Bioinformatics and Integrative Biology, University of Massachusetts, Worcester, MA, USA
[3] Curriculum in Genetics and Molecular Biology, University of North Carolina, Chapel Hill, USA
[4] College of Medicine, University of Malawi, Blantyre, Malawi
[5] Department of Clinical Sciences, Liverpool School of Tropical Medicine, Liverpool, United Kingdom
[6] Centre d'Etude et de Recherche sur le paludisme associé à la Grossesse et à l'Enfance, Université d'Abomey-Calavi, Cotonou, Benin
[7] COMUE Sorbonne Paris Cité, Université Paris Descartes, Paris, France
[8] UMR216 - MERIT, Institut de Recherche pour le Développement, Paris, France
[9] Division of Transfusion Medicine, Department of Medicine, University of Massachusetts, Worcester, MA, USA; Program in Bioinformatics and Integrative Biology, University of Massachusetts, Worcester, MA, USA
[10] Division of Infectious Diseases and International Health and Duke Global Health Institute, Duke University Medical Center, Durham, NC, USA
[11] Division of Infectious Diseases, University of North Carolina School of Medicine, Chapel Hill, NC, USA; Department of Epidemiology, Gillings School of Global Public Health, University of North Carolina, Chapel Hill, NC, USA

*Corresponding Author
Jaymin C. Patel, PhD, MPH
Phone: 919-843-7354
Email: jaymin.patel@unc.edu

## Supplementary Methods

*Patient Samples:* In Malawi, samples were obtained from a randomized controlled trial aimed to assess the efficacy of intermittent screening and treatment in pregnancy (ISTp) with Dihydroartemisinin-Piperaquine (DP)[51]. The trial was conducted between 2010 and 2013 where 1,873 HIV-negative pregnant women were randomized to receive either at least three doses of intermittent preventive treatment in pregnancy (IPTp) with Sulfadoxine-Pyrimethamine (SP) or at least three screenings with a rapid diagnostic test (RDT) and subsequent treatment of RDT-positive cases with DP. In Benin, samples were acquired from a prospective cohort study conducted to quantify the effects of PAM and investigate immunological responses to malaria infection during pregnancy [52]. 1,037 pregnant women were enrolled starting in June 2008 and the last delivery occurred in September 2010. Follow-up in both studies was done during routine antenatal care visits. Women in both studies were enrolled after obtaining signed informed consent.

*P. falciparum* was detected by PCR in the placental blood samples of 281(18.8%) women in Malawi and 175(27.6%) women in Benin; of these, 281 (100%) and 126 (72%) samples were available from Malawi and Benin, respectively, to attempt amplification of ID1-DBL2x.

*Chelex DNA Extraction:* Briefly, DBS were incubated overnight at 4 °C in 10% saponin. After incubation 20% Chelex was added to each sample and incubated at 95°C for 12 minutes. Genomic DNA in the solution was aspirated to separate the Chelex beads and stored at -20°C.

*Column based DNA extraction:* Briefly, packed RBCs were digested with Proteinase K and incubated with ethanol at 56°C for ten minutes. DNA was separated from the lysate via purification column, eluted in water, and stored at -20°C.

*PCR design and reaction conditions:* As amplifying a long hypervariable fragment of *var2csa* from DBS proved to be challenging even after using lower extension temperatures during PCR[72,73], we developed a hemi-nested PCR amplification strategy to amplify the 1.6 kb ID1-DBL2x region of *var2csa* from DNA extracted from DBS. First, all publicly-available gene sequences of *var2csa* from GenBank and PlasmoDB were downloaded and aligned in MEGA6.0 using MUSCLE alignment [55]. Published primers targeting the 1.6 kb region [31] were superimposed on the alignment to ensure that the primers were indeed in a conserved region. An outer reverse primer was designed for the first round of the hemi-nested PCR (Table S1). Barcodes were attached to the forward and reverse primers for the second round PCR (Table S2). The second round of hemi-nested PCR was a designed to be a touchdown PCR in order to increase the specificity of the DNA amplification.

The PCR reaction mix for the first round contained: 2.5µl of Roche Hi-Fi buffer (Roche, Basel, Switzerland), 0.5 µl of 10 mM dNTPs, 20 µM of each primer, 1.2 µl of 25 mM MgCl2, 0.25 µl of Roche Hi-Fi DNA polymerase, 1.25 µl of DMSO, and 5 µl of DNA template in a 25 µl reaction. The first round PCR cycling conditions were as follows: 95°C for 2m, 35 cycles of 95°C for 30s, 52°C for 30s, and 72°C for 3min, and a final extension of 72°C for 7m. PCR products from the primary round were then used as DNA template for the second round PCR. The PCR reaction mix for the second round contained: 2.5µl of Roche Hi-Fi buffer, 0.5 µl of 10 mM dNTPs, 20 µM of each barcoded primer, 0.25 µl of Roche Hi-Fi DNA polymerase, 1.25 µl of DMSO, and 2 µl of primary PCR product in a 25 µl reaction. The second round PCR cycling conditions were as follows: 95°C for 2m, 15 cycles of 95°C for 30s, 67-52°C for 30s (-1°C per cycle during first 15 cycles, then 52°C for 25 cycles), and 72°C for 3m, and a final extension of 72°C for 7m. All second round PCR products were visualized on a 1% agarose gel.

*Generation of mixtures for validation study:* Stocks of genomic DNA from seven parasite lines (3D7, FCR3, 7G8, DD2, K1, RO33, and V1/S) received from BEI resources (formerly known as MR4, Manassas, VA), were first quantified using the *P. falciparum* tubulin gene through droplet digital PCR (ddPCR) on the Biorad QX200 digital droplet PCR system (BioRad, Hercules, CA). The stocks were then diluted 1:50 using water. Concentration of template DNA for the seven parasite lines ranged from 95 to 627 copies/*µl.* Each parasite line in the dilution series was then mixed together in varying frequencies (1-50%) in five pools. The pools were then subjected to our PCR amplification strategy using dual barcoded primers and deep-sequenced on one PacBio SMRT cell.

*Bioinformatics analyses for haplotype generation:* First, sequences sharing the same barcodes were clustered using a k-mer clustering algorithm. For each sequence, all its k-mers were indexed. When indexing an entire sequence for all k-mers of a given length, sub-sequence strings were selected starting with the first position. The total number of k-mers of a given length for a sequence is the length of the sequence minus the k-mer length plus 1. We defined a k-mer similarity score as the total number of shared k-mers between two sequences divided by the total number of possibly shared k-mers between the two sequences. A k-mer similarity score of 0 indicated no shared k-mers, while a k-mer similarity score of 1 complete k-mer sharing. To cluster the pacbio sequences we calculated a k-mer similarity score at k-mer sizes of 2, 3, and 4. Second, after within-PCR replicate (same barcode) clustering, between-PCR replicate (same samples) or population clustering was performed to determine the similarity between technical replicates from each sample, identify different variants of the ID1-DBL2x-ID2 region, and estimate the frequencies of each variant. Identification of variants and estimation of their frequencies was done within each sample as well as between samples at a population level. Haplotypes from technical PCR duplicates from each patient sample were only accepted when they appeared in both replicates. The haplotype frequencies were calculated as the average of the frequencies in the two replicates. The end result of k-mer clustering yielded one or more phased consensus sequences for each woman present in the study population **(**Figure S1). All variants were initially aligned in MEGA6 using MUSCLE alignment. Due to a large number of insertions and deletions and polyA repeats, we manually curated the alignment to improve the alignment output from MEGA6

*Characterizing species richness and within population diversity:* To characterize the genetic diversity of the ID1-DBL2x region among field isolates, we calculated species richness and other measures of alpha (within-population) diversity. We then compared these measures between the two countries and between women of different gravidity. To estimate species richness of *var2csa*, we calculated rarefaction curves in Malawi and Benin as well as by gravidity in the combined population. Because observed species richness is highly sensitive to both sample size and the diversity of the marker being used, a simple ratio of species per sampling unit may distort richness values. Hence rarefaction curves were calculated that take into account how sampling was conducted. Rarefaction curves of ID1-DBL2x variants were calculated in EstimateS [56]. Each rarefaction curve was bootstrapped 1000 times with replacement to generate 95% confidence intervals. Since *var2csa* is highly diverse and we were likely to find many more variants of *var2csa* through next-generation sequencing, we extrapolated the rarefaction curves by 100 additional samples to predict how species richness would change if our sample size were larger.

We used several metrics to estimate the within population (alpha) diversity of ID1-DBL2x. We calculated expected heterozygosity ($H_e$), Shannon index (H’), abundance coverage estimator (ACE), incidence coverage estimator (ICE), and Chao richness estimators [59-63]. Expected

heterozygosity ($H_e$) was calculated in R using the adegenet and pegas packages [57,58] at each locus within each country and within women of differing gravidities All Estimates of $H_e$ were then bootstrapped 1000 times to estimate precision and test if $H_e$ was statistically different by country or by gravidity. Additionally, to examine how genetic diversity in the variants affected protein sequences, we calculated $H_e$ at each amino acid position among variants in Malawi and Benin from translated protein sequences. Shannon index, ACE, ICE, and the Chao estimates by country and gravidity were calculated on the genetic sequences in EstimateS [56] and bootstrapped 1000 times. Statistically significant differences in alpha diversity metrics between different groups were tested using Kruskal–Wallis test. An α of 0.05 was determined *a priori* to test for significant differences.

*Phylogenetic Analyses:* Using genetic sequences from the haplotypes, we constructed phylogenetic trees using the maximum composite likelihood method in MEGA6. Genetic distances in the maximum composite likelihood method were calculated using the Tamura-Nei model [68]. The maximum composite likelihood phylogenetic trees were bootstrapped 1000 times to compute branch support and provide precision in the differences observed in the trees. All phylogenetic trees were visualized using the APE package for R [69].

*Between population (β) diversity:* To characterize genetic relatedness among the ID1-DBL2x variant populations between Malawi and Benin as well as between the different clades, we calculated Wright's fixation index ($F_{ST}$) [64]. The $F_{ST}$ index quantifies genetic relatedness based on allele frequencies among population and ranges from 0 to 1, where 0 signifies a panmictic population and 1 signifies completely differentiated populations. The $F_{ST}$ analyses were performed using a sliding window approach to identify regions along the vaccine target that would account for the genetic relatedness between countries and clades. We performed a principal coordinate analysis (PCoA) to assess the genetic relatedness between population of variants from different countries, gravidities, and clades identified by phylogenetic clustering. $F_{ST}$ values were used as the genetic distance matrix for the PCoA. The top coordinates explaining the most amount of variation were used to visualize the genetic relatedness between the clades.

We calculated nucleotide diversity (π) and Tajima's D test [65] for the entire ID1-DBL2x region as well as with a sliding window approach to assess localized selection pressures acting upon vaccine target [66]. Tajima's D was calculated on ID1-DBL2x variant populations by country and major clades. All measures of genetic relatedness and selection ($F_{ST}$, PCoA, π, Tajima's D) were calculated in R using the adegenet [57] and PopGenome [67] packages.

REFERENCES:


72 Lopez-Barragan, M. J. *et al.* Effect of PCR extension temperature on high-throughput sequencing. *Mol Biochem Parasitol* **176**, 64-67, doi:10.1016/j.molbiopara.2010.11.013 (2011).

73 Su, X. Z., Wu, Y., Sifri, C. D. & Wellems, T. E. Reduced extension temperatures required for PCR amplification of extremely A+T-rich DNA. *Nucleic Acids Res* **24**, 1574-1575 (1996).

Table S1: ID1-DBL2x hemi-nested PCR primer sequences

| Primer Name | 5'→3' |
|---|---|
| ID1-F* | GATCCTTATTCCGCAGAATA |
| CIDR-R_Heminested | TTTCTTTGTTCCACTGTTCAAA |
| CIDR-R* | GTCGTGTATGTTGTCCA |

*Bordbar et. al, 2014

**Table S2.** Barcoded primer sequences for ID1-DBL2x PCR assay

| Forward Primer ID | Barcode | Forward Barcoded primer (5'→3') | | Reverse Primer ID | Barcode | Reverse Barcoded primer (5'→3') |
|---|---|---|---|---|---|---|
| Id1-F-MID22 | TACGAGTATG | TACGAGTATGGATCCTTATTCCGCAGAATA | | CIDR-R-MID22 | CATACTCGTA | CATACTCGTAGTCGTGTATGTTGTCCA |
| Id1-F-MID23 | TACTCTCGTG | TACTCTCGTGGATCCTTATTCCGCAGAATA | | CIDR-R-MID23 | CACGAGAGTA | CACGAGAGTAGTCGTGTATGTTGTCCA |
| Id1-F-MID24 | TAGAGACGAG | TAGAGACGAGGATCCTTATTCCGCAGAATA | | CIDR-R-MID24 | CTCGTCTCTA | CTCGTCTCTAGTCGTGTATGTTGTCCA |
| Id1-F-MID25 | TCGTCGCTCG | TCGTCGCTCGGATCCTTATTCCGCAGAATA | | CIDR-R-MID25 | CGAGCGACGA | CGAGCGACGAGTCGTGTATGTTGTCCA |
| Id1-F-MID26 | ACATACGCGT | ACATACGCGTGATCCTTATTCCGCAGAATA | | CIDR-R-MID26 | ACGCGTATGT | ACGCGTATGTGTCGTGTATGTTGTCCA |
| Id1-F-MID27 | ACGCGAGTAT | ACGCGAGTATGATCCTTATTCCGCAGAATA | | CIDR-R-MID27 | ATACTCGCGT | ATACTCGCGTGTCGTGTATGTTGTCCA |
| Id1-F-MID28 | ACTACTATGT | ACTACTATGTGATCCTTATTCCGCAGAATA | | CIDR-R-MID28 | ACATAGTAGT | ACATAGTAGTGTCGTGTATGTTGTCCA |
| Id1-F-MID29 | ACTGTACAGT | ACTGTACAGTGATCCTTATTCCGCAGAATA | | CIDR-R-MID29 | ACTGTACAGT | ACTGTACAGTGTCGTGTATGTTGTCCA |
| Id1-F-MID30 | AGACTATACT | AGACTATACTGATCCTTATTCCGCAGAATA | | CIDR-R-MID30 | AGTATAGTCT | AGTATAGTCTGTCGTGTATGTTGTCCA |
| Id1-F-MID31 | AGCGTCGTCT | AGCGTCGTCTGATCCTTATTCCGCAGAATA | | CIDR-R-MID31 | AGACGACGCT | AGACGACGCTGTCGTGTATGTTGTCCA |
| Id1-F-MID32 | AGTACGCTAT | AGTACGCTATGATCCTTATTCCGCAGAATA | | CIDR-R-MID32 | ATAGCGTACT | ATAGCGTACTGTCGTGTATGTTGTCCA |
| Id1-F-MID33 | ATAGAGTACT | ATAGAGTACTGATCCTTATTCCGCAGAATA | | CIDR-R-MID33 | AGTACTCTAT | AGTACTCTATGTCGTGTATGTTGTCCA |
| Id1-F-MID34 | CACGCTACGT | CACGCTACGTGATCCTTATTCCGCAGAATA | | CIDR-R-MID34 | ACGTAGCGTG | ACGTAGCGTGGTCGTGTATGTTGTCCA |
| Id1-F-MID35 | CAGTAGACGT | CAGTAGACGTGATCCTTATTCCGCAGAATA | | CIDR-R-MID35 | ACGTCTACTG | ACGTCTACTGGTCGTGTATGTTGTCCA |
| Id1-F-MID36 | CGACGTGACT | CGACGTGACTGATCCTTATTCCGCAGAATA | | CIDR-R-MID36 | AGTCACGTCG | AGTCACGTCGGTCGTGTATGTTGTCCA |
| Id1-F-MID37 | TACACACACT | TACACACACTGATCCTTATTCCGCAGAATA | | CIDR-R-MID37 | AGTGTGTGTA | AGTGTGTGTAGTCGTGTATGTTGTCCA |
| Id1-F-MID38 | TACACGTGAT | TACACGTGATGATCCTTATTCCGCAGAATA | | CIDR-R-MID38 | ATCACGTGTA | ATCACGTGTAGTCGTGTATGTTGTCCA |
| Id1-F-MID39 | TACAGATCGT | TACAGATCGTGATCCTTATTCCGCAGAATA | | CIDR-R-MID39 | ACGATCTGTA | ACGATCTGTAGTCGTGTATGTTGTCCA |

**Table S3:** Characteristics of PCR amplified and unamplified samples

| Group | Amplified (n=101) | Not Amplified (n = 315) | P values |
|---|---|---|---|
| Maternal Age | | | |
| Mean (SD) | 22.5 (5.5) | 24.5 (6.2) | 0.82 |
| | | | |
| Gestational Age | | | 0.83 |
| Mean (SD) | 38.1(2) | 38.87(1.9) | |
| | | | |
| Gravidity, n (%) | | | |
| Primigravid | 39(38.6) | 97 (30.8) | 0.15 |
| Secundigravid | 29(28.7) | 89 (28.3) | 0.99 |
| Multigravid | 31(32.7) | 129 (41.0) | 0.16 |

**Table S4.** Nucleotide diversity (π) and Tajima's D. The fragment size assessed in this analysis was ~1600 nucleotides. The Tajima's D test statistic can indicate whether a nucleotide sequence is under directional selection ($D<0$), genetic drift ($D=0$), or balancing selection ($D>0$).

| Group | n* | π** | Segregating sites | Tajima's D |
|---|---|---|---|---|
| All | 152 | 0.105 | 470 | 1.781 |
| Malawi | 57 | 0.096 | 415 | 1.976 |
| Benin | 95 | 0.113 | 460 | 1.699 |
| | | | | |
| Primigravid | 63 | 0.099 | 459 | 1.183 |
| Secundigravid | 49 | 0.11 | 451 | 1.244 |
| Multigravid | 53 | 0.115 | 458 | 1.467 |

*number of haplotypes
**nucleotide diversity

**Table S5.** Within-population diversity of ID1-DBL2x populations. The metrics which do not compensate for sampling ($H_e$ and $H'$) showed no difference between the sites while the metrics that do (ACE, ICE, and Chao), as predicted, demonstrate significantly greater diversity in Benin.

| Group | n* | $H_E$ (SD) | Shannon (SD) | ACE (SD) | ICE (SD) | Chao1 (SD) | Chao2 (SD) |
|---|---|---|---|---|---|---|---|
| All | 152 | 0.266 (0.027) | 4.5 (0.08) | 157.12 (21.96) | 157.36 (22.08) | 149.51 (16.65) | 149.31 (16.59) |
| Malawi | 95 | 0.307 (0.019) | 4.04 (0.13) | 104.19 (19.32) | 104.51 (19.5) | 100.21 (15.31) | 99.91 (15.19) |
| Benin | 57 | 0.298 (0.037) | 3.98 (0.02) | 257.3 (23.87) | 259.7 (24.09) | 201.81 (64.7) | 200.8 (64.16) |
| | | | | | | | |
| Primigravid | 63 | 0.274 (0.027) | 3.56 (0.09) | 65.06 (16.13) | 65.29 (16.33) | 61.84 (12.2) | 61.61 (12.09) |
| Secundigravid | 49 | 0.299 (0.032) | 3.34 (0.13) | 55.97 (18.49) | 56.29 (18.8) | 52.72 (12.82 | 52.42 (12.65) |
| Multigravid | 53 | 0.300 (0.035) | 3.41 (0.12) | 57.91 (15.59) | 57.71 (15.85) | 57.25 (13.65) | 56.94 (13.47) |

*number of haplotypes

**Table S6:** Associations between ID1-DBL2x vaccine clades (3D7 & FCR3) and small-for-gestational age (SGA) and low birthweight (LBW)

| | **Crude** | | | | **Adjusted**** | | |
|---|---|---|---|---|---|---|---|
| **SGA** | **OR*** | **95% CI** | | | **OR*** | **95% CI** | |
| **Pooled** | 2.45 | 0.67 | 8.94 | | 3.65 | 1.00 | 13.38 |
| | | | | | | | |
| **Malawi** | 3.46 | 0.59 | 20.20 | | 5.21 | 0.77 | 35.41 |
| | | | | | | | |
| **Benin** | 2.67 | 0.25 | 28.44 | | 2.98 | 0.27 | 32.48 |
| | | | | | | | |
| **LBW** | | | | | | | |
| **Pooled** | 7.86 | 1.89 | 32.69 | | 5.41 | 1.00 | 29.52 |
| | | | | | | | |
| **Malawi** | 19.09 | 2.16 | 169.09 | | 13.49 | 1.01 | 179.69 |
| | | | | | | | |
| **Benin** | 1.50 | 0.10 | 23.07 | | 0.94 | 0.01 | 70.42 |

Reference group = FCR3 clade

** Adjusted for country (pooled analysis only) and parity using inverse probability weights (IPW)

**Figure S1.** Schematic representation of the bioinformatics clustering pipeline employed in this study. Pools of amplified ID1-DBL2x from individual women in technical replicates using barcoded primers were sequenced on PacBio CCS platform. Reads were clustering using a k-mer algorithm to identify unique ID1-DBL2x variants in the study population.

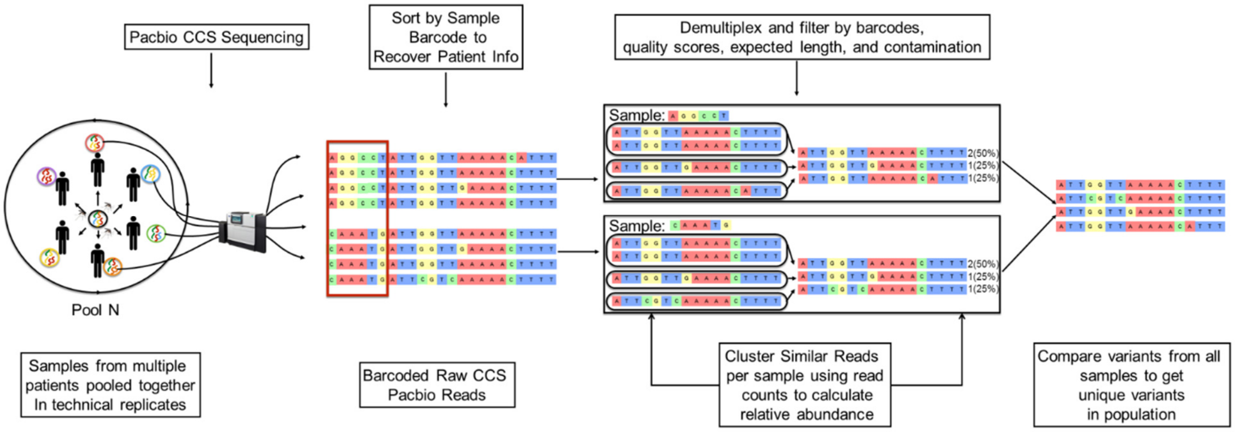

**Figure S2.** Comparison of expected haplotype values to two analysis methods for validation. Five artificial mixtures of parasite strains were generated as described in the Supplementary Information. The first column in each group represents the “expected” frequencies of haplotypes based upon the mixtures created. The second column shows haplotype frequencies predicted by mapping each PacBio read back to its closest reference parent based on Sanger sequencing of the gene. The last column shows the haplotype frequencies determined by the k-means clustering pipeline.

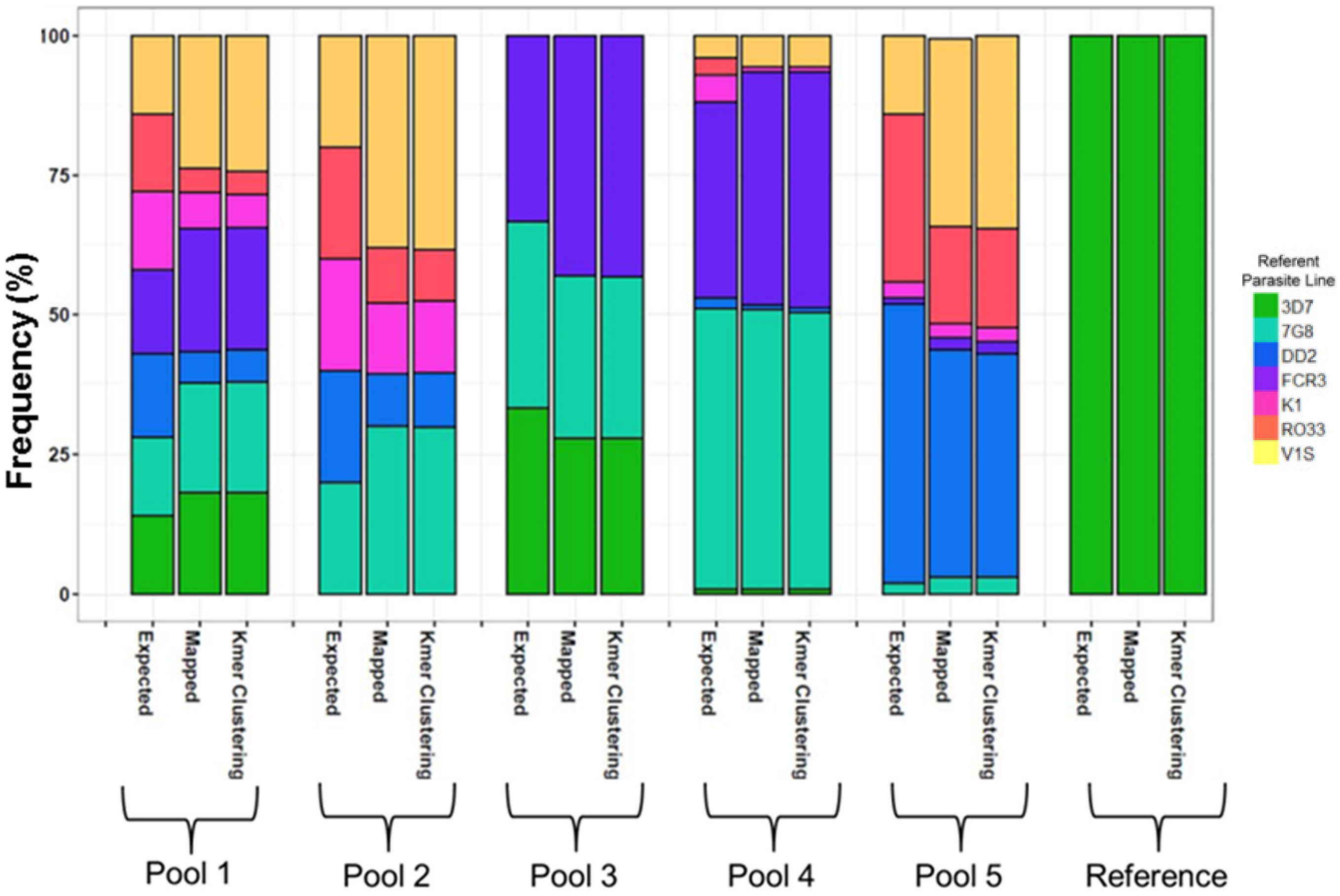

**Figure S3.** Scatterplot of expected frequencies vs. observed frequencies (employing k-mer clustering) of ID1-ID2x haplotypes in mixtures of reference parasite lines.

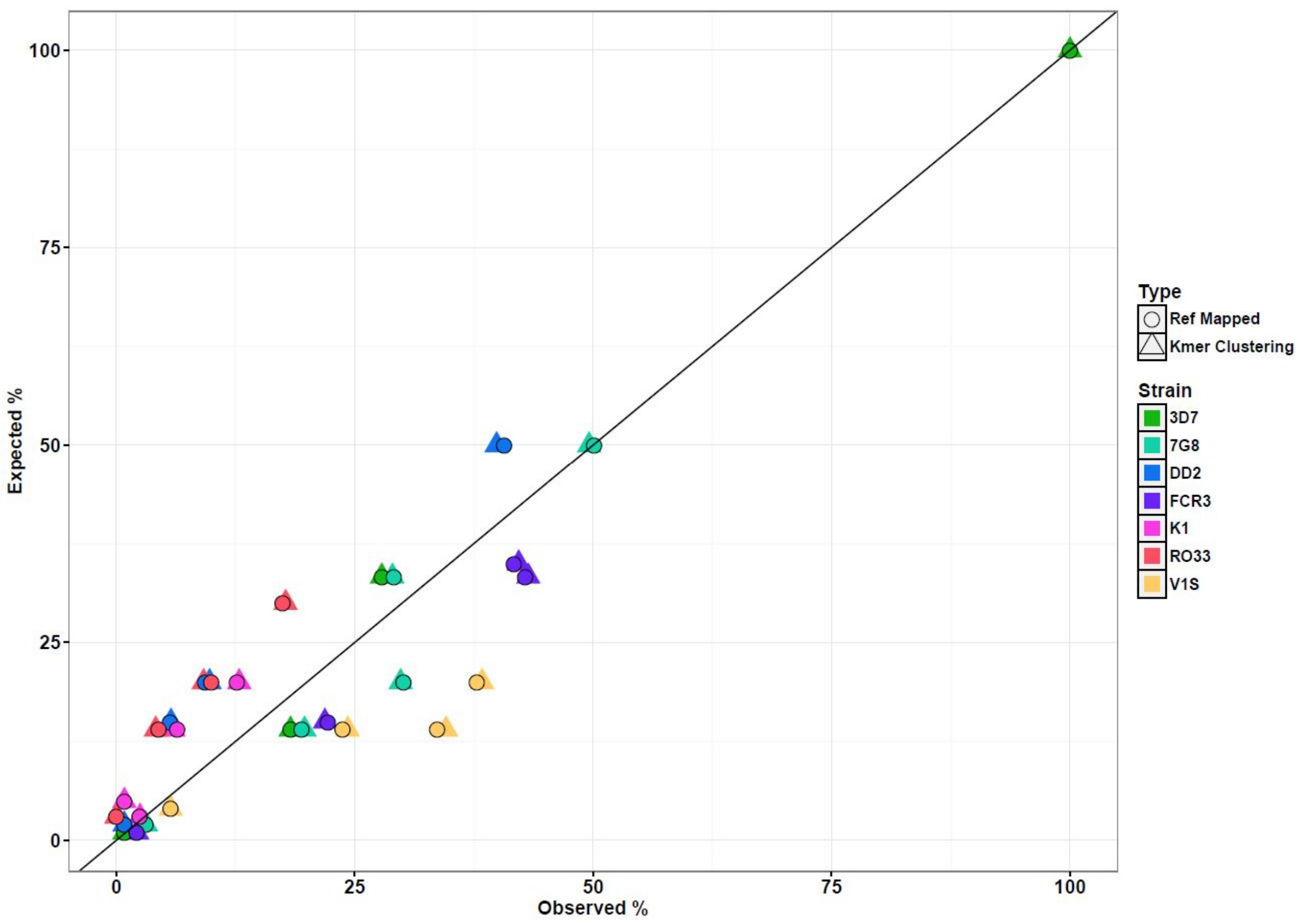

**Figure S4.** Read Filtering For Clinical Samples.

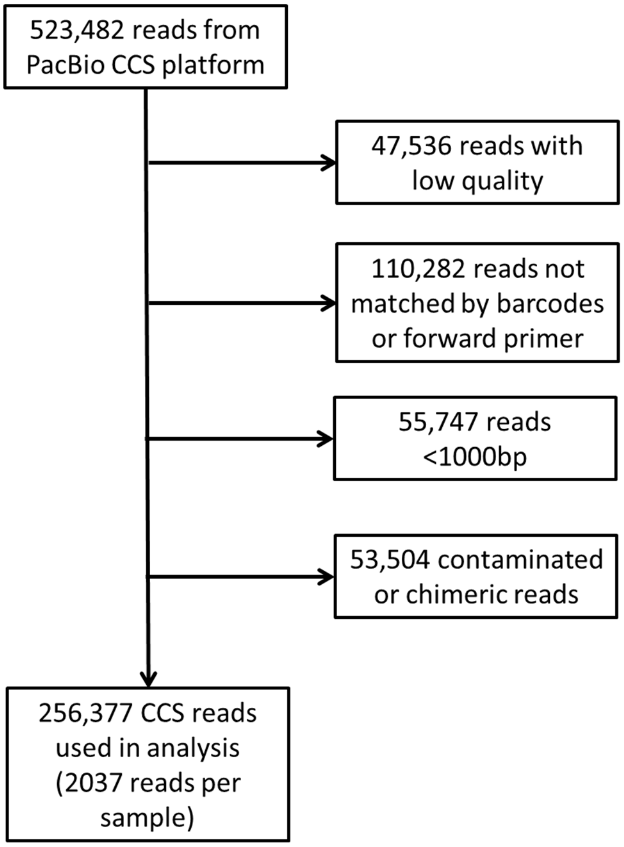

**Figure S5.** Expected heterozygosity ($H_e$) at each amino acid position along the ID1-DBL2x region among variants populations in Malawi (blue) and Benin (red). $H_e$ at the amino acid level along the ID1-DBL2x fragment showed regions of high diversity separated by conserved regions. A short region of higher amino acid diversity is seen in the ID1 region in Benin and is driven by the parasites from the unique clusters from that country.

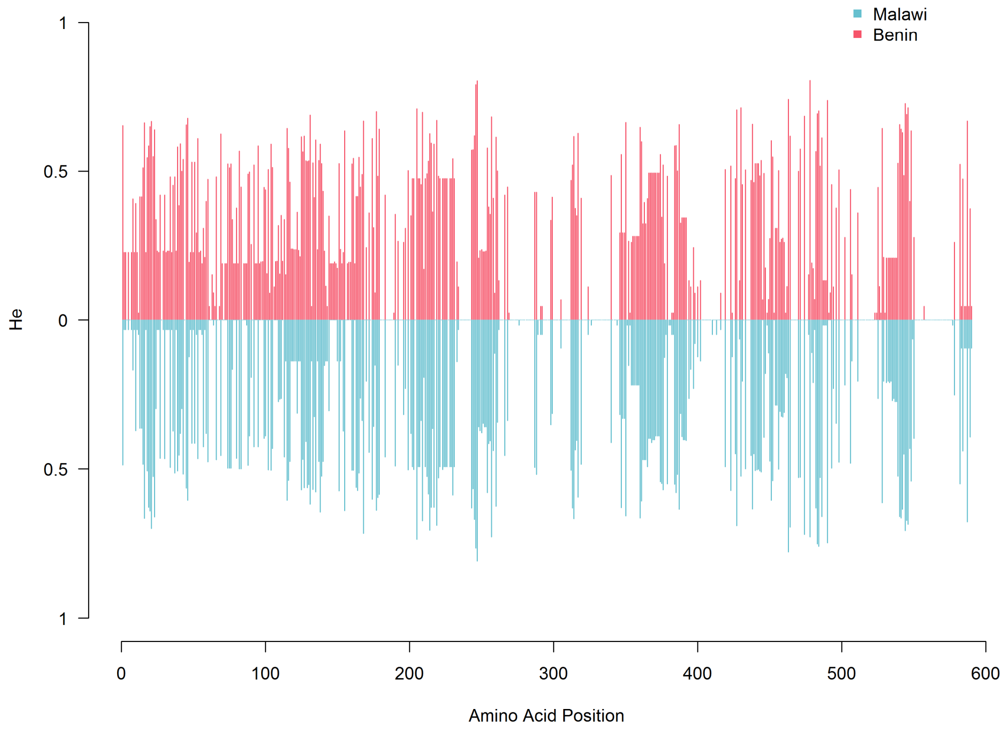

**Figure S6.** $F_{ST}$ values using a sliding window approach (window size = 10bp, step size = 10bp) across the 1.6 kb ID1-DBL2x region of *var2csa*. $F_{ST}$ values were calculated between the two major vaccine clades (3D7 and FCR3). $F_{ST}$ values range from 0 to 1, where 0 signifies a genetically identical population and 1 signifies completely differentiated populations. Sliding window $F_{ST}$ analyses show that scanning across the region, the parasite populations in the two clades are very similar except for a ~100 bp region (highlighted in gray) where the two populations differ substantially.

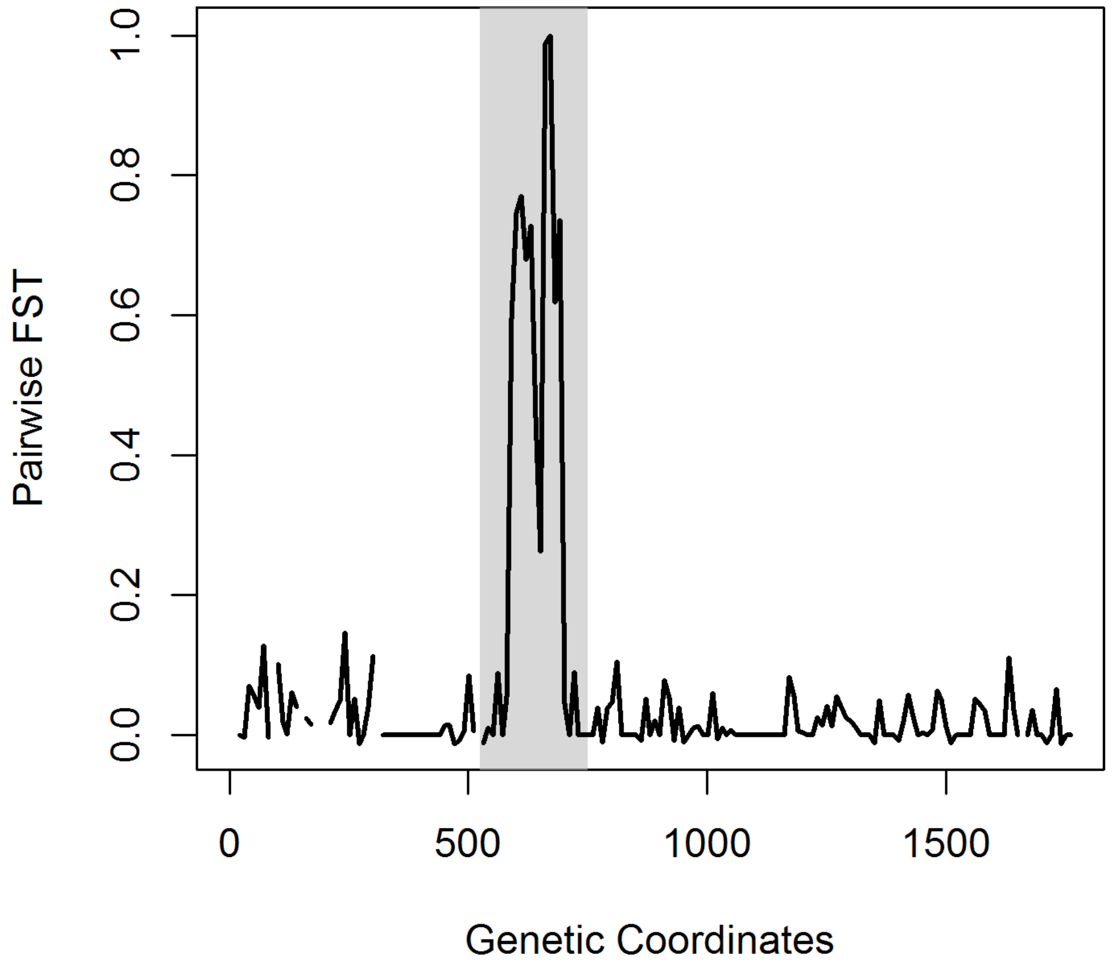

**Figure S7**. Tajima's D using a sliding window approach (window size = 100bp, step size = 25bp) across the 1.6kb ID1-DBL2x region of *var2csa*. (A) Tajima's D calculated for ID1-DBL2x populations in Malawi and Benin and (B) 3D7 and FCR3 clades. Tajima D values less than 0 indicate directional selection, equal to 0 indicate genetic drift, and greater than 0 indicate balancing selection.

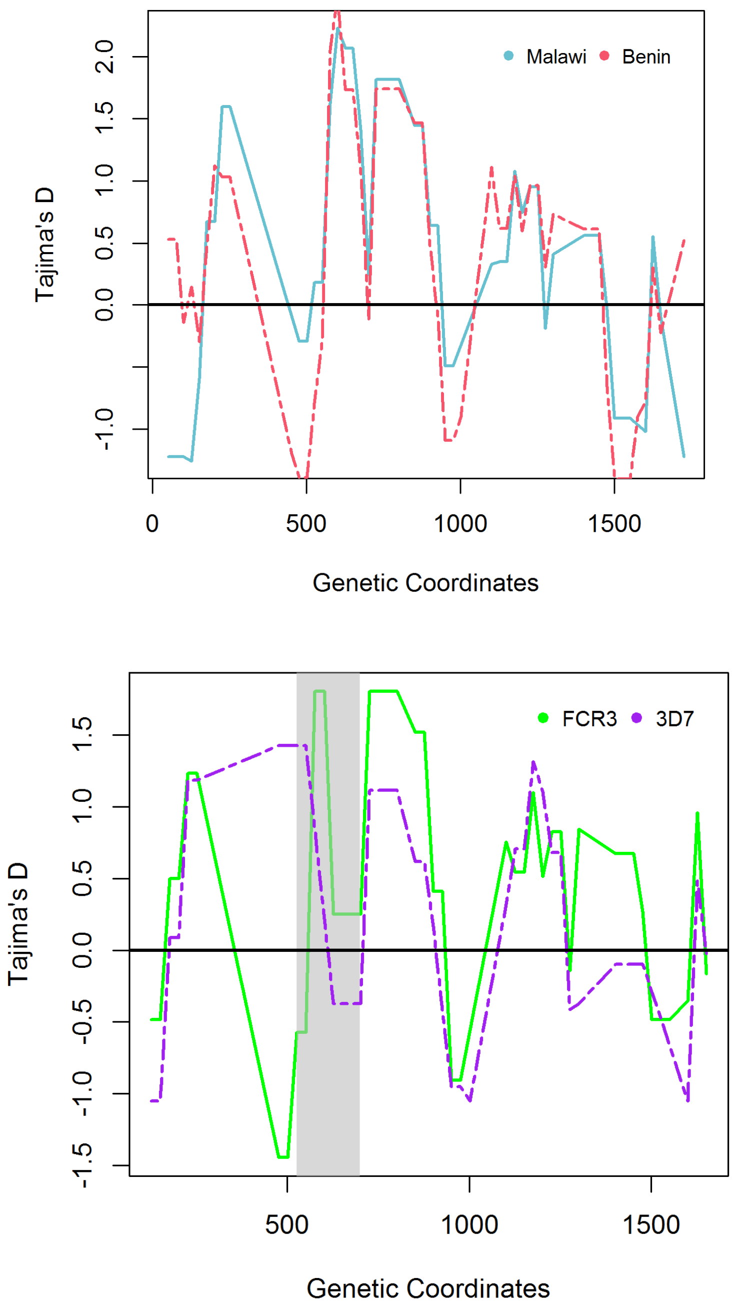

**Figure S8**: Principal Coordinate Analysis (PCoA) of ID1-DBL2x variants in the study population. (A) PCoA plot using principal coordinates 1 and 2 (PC1 & PC2) (B) PCoA plot using principal coordinates 1 and 3 (PC1 & PC3). Each dot represents the unique ID1-DBL2x variants colored according to the clade. The axes indicate which coordinates are being plotted and the percentage of variation explained by that particular axis.

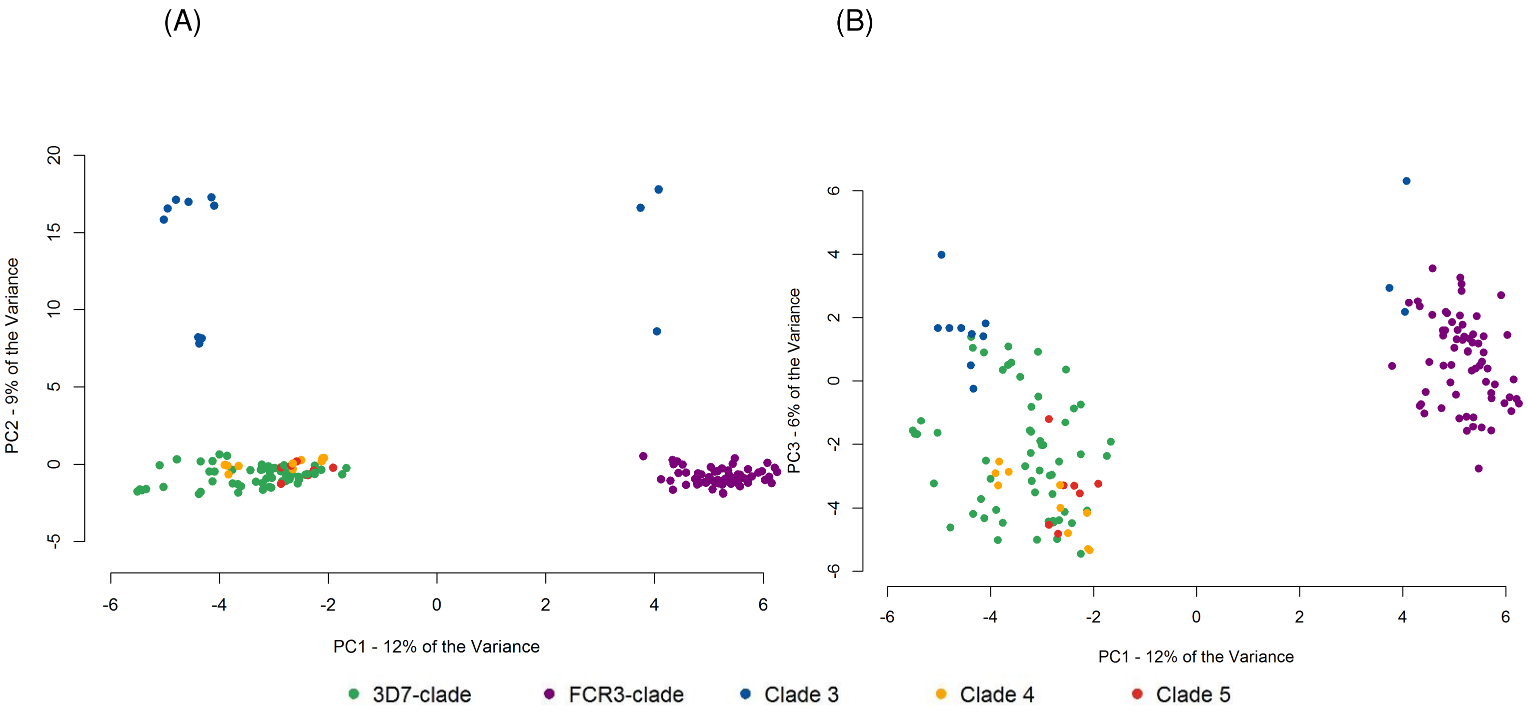

Supplement: Supplementary file 1 — Supplementary Information [file 41598_2017_4737_MOESM1_ESM.pdf]
